# Evaluating Satisfaction and Self-Confidence among Nursing Students in Clinical Simulation Learning

**DOI:** 10.3390/nursrep14020078

**Published:** 2024-04-25

**Authors:** Sara Moreno-Cámara, Henrique da-Silva-Domingues, Laura Parra-Anguita, Belén Gutiérrez-Sánchez

**Affiliations:** Department of Nursing, Faculty of Health Sciences, University of Jaén, 23071 Jaén, Spain; smcamara@ujaen.es (S.M.-C.); lparra@ujaen.es (L.P.-A.); bgutierr@ujaen.es (B.G.-S.)

**Keywords:** nursing education research, family nursing, simulation, high-fidelity simulation training, satisfaction, self-confidence

## Abstract

Background: Clinical simulation is effective in nursing student education, fostering autonomous learning and critical skill development in safe environments. This method is adaptable to dynamic educational approaches and integrates technology. Satisfaction and self-confidence are key elements in its evaluation. The general objective of this research was to describe the levels of satisfaction and self-confidence among undergraduate nursing students regarding the use of clinical simulation in the field of family and community nursing. Methods: A cross-sectional descriptive study was conducted at the University of Jaén, Spain, during the 2023/2024 academic year. Data on sociodemographic aspects, satisfaction, and self-confidence were collected using a validated instrument. The statistical analysis included central measures, dispersion, and frequencies, with confidence intervals. Results: The study involved 96 students in scenario 1 (family assessment) and 97 in scenario 2 (family intervention), with the majority being women. In scenario 1, the mean satisfaction score was 4.38 out of 5, and self-confidence was scored 4.44 out of 5. Prior preparation time correlated significantly with higher levels of satisfaction and self-confidence. In scenario 2, the mean scores were slightly higher but not statistically significant. Conclusions: Our study demonstrated high levels of satisfaction and self-confidence among nursing students following clinical simulations. Prior preparation was associated with better outcomes, and the quality of the simulation positively impacted the results.

## 1. Introduction

Driven by the scientific evidence of the efficacy of innovative teaching methods, nursing education is undergoing a transformation towards more dynamic approaches, with a particular emphasis on simulations [1]. Alongside the development of simulated clinical scenarios, traditional teaching strategies such as expository lectures, dialogic sessions, and skills training continue to be maintained and refined [2]. Furthermore, the integration of technology in teaching emerges as an essential tool to enhance learning and cultivate competencies in nursing students.

Practical nursing education has seen significant growth through the continuous replication of techniques and immersion in scenarios where students engage in hands-on practice and refine their skills by interacting with patient-actors, simulators, and peers in training [2]. However, some studies show a significant skill gap in future health professionals, especially in the field of primary health care and nursing interventions for families [3,4]. In this sense, it is necessary to reinforce the training of these students by using more effective training aligned with the current demands of professional practice [5,6].

In this respect, clinical simulation (CS) has proven to be effective in nursing student education [6]. According to the National League for Nursing (NLN), simulation is defined as the replication of essential elements of real scenarios in a simulated environment to enhance students’ understanding and management of such situations in actual clinical practice [5]. This training strategy enables students to learn autonomously through active practice and the development of critical skills [6,7,8], engaging them in a safe environment closely resembling real-life situations [7,9,10]. This alternative teaching method facilitates the integration of knowledge and skills in a structured and meaningful manner [5,6].

The literature demonstrates that CS is a valuable educational tool, yielding excellent results in reinforcing the integration of theory and practice and deepening acquired knowledge, while also contributing to the practical training of future nursing professionals [11]. Additionally, it serves as an effective means of learning and evaluating both technical and non-technical skills, offering students a guided initial experience that simulates real aspects of patient assessment, diagnosis, and care [7,12].

Indeed, CS enables students to develop skills and knowledge safely within a learning environment closely resembling clinical practice [13]. Students appreciate this method as it allows them to bridge theory with practice and engage in situations akin to those encountered in clinical settings, thereby requiring them to act, behave, and think as they would in real scenarios [14].

The preparation of simulation experiences should be grounded in the best available scientific evidence to ensure the optimal impact on the students’ training. Therefore, it is essential to measure variables related to this experience to enhance this teaching–learning method. A meta-review [6] evaluated and synthesized the evidence on the influence of CS on undergraduate nursing students. Among the five included reviews, a strong association was found between students’ satisfaction with simulation training and improved confidence levels in three studies [6].

Various outcomes associated with this training strategy have been investigated, including simulation involvement, self-efficacy, psychomotor impact, non-technical skills, patient safety, and simulation effectiveness [6]. Furthermore, original research conducted in different countries, including the United States [15], Korea [16], Brazil [17,18], and Spain [19,20] has also reported a positive association between satisfaction and confidence and CS among nursing students.

Satisfaction and self-confidence were highlighted as essential elements for evaluating the effectiveness of educational strategies, emphasizing the need to address these aspects in a reflective manner [21]. Satisfaction, understood as the feeling of pleasure or disappointment derived from comparing performance with personal expectations [22], is highly relevant as it is linked to greater commitment and motivation towards the learning process [1]. Self-confidence is defined as the conviction of possessing the ability to perform a specific task, manifesting the individual’s competence in achieving personal goals [23]. Students who are confident in their abilities not only experience benefits at the individual level, but also contribute to the improvement of quality standards in educational institutions [21].

While the existing literature underscores the positive impact of CS on nursing education, there remains a notable gap in assessing the satisfaction levels, the promotion of self-confidence among students engaged in CS [15], as well as the significant skill deficit in future primary care nurses [3,4]. Thus, the primary objective of this research was to describe the levels of satisfaction and self-confidence among nursing students concerning the use of CS in the field of family and community nursing. Additionally, our secondary objective was to examine the relationship between age, gender, and the duration of preparation prior to the simulation experience and student satisfaction and self-confidence.

## 2. Materials and Methods

### 2.1. Study Design

A cross-sectional study was conducted.

### 2.2. Participants

The study population comprised 130 third-year nursing students enrolled in the Family and Community Nursing III (F&CN III) course at the University of Jaén, Spain, during the 2023/2024 academic year.

### 2.3. Sample

The sampling method employed was non-probabilistic convenience sampling.

#### 2.3.1. Sample Size

Out of the 130 participants comprising the total study population, the sample comprised 96 university students in scenario 1 and 97 university students in scenario 2 (the same students participated in both scenarios).

#### 2.3.2. Inclusion Criteria

The inclusion criteria were course enrollment in the EFyC III course during the 2023/2024 academic year, and voluntary acceptance of participation in the study.

#### 2.3.3. Exclusion Criteria

The exclusion criteria were having a visual impairment that hindered the ability to read the questionnaire, and students who did not agree to participate in the research and did not have internet access on their mobile device at the time of data collection.

This study was approved by the Ethics Committee of the University of Jaén (Reference: JUL.23/1 PRY). All participants (n = 96 students in scenario 1 and n = 97 students in scenario 2) signed the informed consent form and the confidentiality of the results was guaranteed by not collecting any identifying data from the participants.

### 2.4. Clinical Simulation Scenarios

At the curricular level, nursing students engage in subjects related to family and community nursing throughout their training. These subjects aim to equip future professionals with a broad spectrum of knowledge, skills, and abilities to provide care for individuals, families, and communities. Specifically, in the Degree in Nursing program at the University of Jaén, Spain, the F&CN III course focuses on the promotion of self-care through interventions at the family level.

The high-fidelity simulation strategy employed to train the nursing students in family intervention was based on the nursing process [24]. This approach systematically organizes nursing care for patients and encompasses five phases: assessment, diagnosis, planning, implementation (intervention), and evaluation [24]. In the context of CS, the specific phases addressed were the family assessment phase (scenario 1) and the implementation phase (family intervention) (scenario 2).

The simulation experience was conducted in a seminar setting designed to resemble a home environment. The scenario involved the participation of the senior theater group of the University of Jaén, portraying a family caring for an elderly, dependent relative. The script followed by the actors was meticulously directed by the professors of the course.

For the CS, there were four face-to-face practical sessions, with two sessions allocated to scenario 1 and two sessions to scenario 2. Each practical session lasted three hours and accommodated 14 students. During these sessions, the entire group participated in the pre-briefing and debriefing sessions for both scenarios

Simultaneously, at a theoretical level, the nursing intervention was taught in a family setting. During this period, students were required to conduct a family assessment (scenario 1) and a subsequent family intervention (scenario 2) based on the assessment findings. The students were provided with guidelines outlining the objectives and tasks for the practical sessions related to nursing interventions in a family setting.

Before the practical sessions, students were tasked with planning both the assessment and the intervention for the family. They were required to submit a document to the teachers containing the essential details such as a brief description of the family, the selected nursing diagnosis, the developed care plan, a description of the planned intervention along with its objectives and proposed interventions, the materials and resources to be used, and the anticipated evaluation timeline for the intervention.

### 2.5. Measures

Sociodemographic variables were collected through an ad hoc questionnaire. Satisfaction and self-confidence variables were assessed using the “Student Satisfaction and Self-Confidence in Learning” questionnaire developed by the National League for Nursing (NLN) [25]. For this study, we used the version of the instrument validated for the Spanish population [26]. This instrument comprises 13 self-administered items, measuring students’ satisfaction with instructional methods, learning materials, and instructors (5 items), as well as their self-confidence in learning (8 items). The responses are recorded on a Likert-type scale ranging from 1 (strongly disagree) to 5 (strongly agree). The total score ranges from 13 to 65 points, with scores for each construct expressed on a scale from 1 to 5. The instrument demonstrates a high level of internal consistency, with a Cronbach’s alpha of 0.90 for both the original version [25] and the Spanish translated version [26]. In previous studies, the Cronbach’s alpha for the satisfaction subscale was 0.94 [25], 0.71 [27], and 0.97 [16]. For the self-confidence subscale, the Cronbach’s alpha obtained was 0.87 [25], 0.70 [27], and 0.87 [16].

### 2.6. Data Collection

Data collection took place in November 2023, following the implementation of the CS in the practical sessions. Prior to administering the questionnaire, the participants were provided with an explanation of the research’s purpose and procedures. An emphasis was placed on the voluntary nature of their participation and the confidentiality of the entire process.

### 2.7. Data Analysis

The descriptive analysis of the quantitative variables involved calculating measures of central tendency and dispersion, while qualitative variables were analyzed using frequencies and percentages, along with their respective confidence intervals (CIs). The normality of the variables was tested with the Kolmogorov–Smirnov test. To examine differences between sex, age (dichotomized), and previous preparation time of the participants (dichotomized), Student’s *t*-test and Cohen’s D statistic were employed.

The analyses were conducted using SPSS Statistics 23 and EpiDat 4.2.

## 3. Results

### 3.1. Sample Description

In scenario 1 (family assessment), the total sample comprised 96 students, with 87% being women and the mean age being 24.05 years. Regarding preparation time prior to practice, 49% dedicated between 2 and 4 h of study.

In scenario 2 (family intervention), the total sample consisted of 97 students, with 87% being women and the mean age being 23.63 years. In terms of preparation time prior to practice, 46% dedicated between 2 and 4 h of study time. The descriptive data of the samples are summarized in Table 1.

### 3.2. Scenario 1 (Family Assessment)

In scenario 1 (family assessment), the mean total score of the instrument was 57.45 (range: 13–65), with a standard deviation of 10.37. For the satisfaction construct, the mean was 4.38, with a standard deviation of 0.85. Notably, 62% of the students expressed complete agreement with the statement “The simulation provided me with a variety of teaching materials and activities that favored my learning in the clinical curriculum”. Similarly, 58% of the students expressed total agreement with the statement “The teaching methods used in this simulation were helpful and effective”. The aspect least valued by the students was the statement “The teaching materials used in this simulation were very motivating and helped me learn” with a mean score of 4.29 and a standard deviation of 0.99.

In the self-confidence construct, the mean was 4.44, with a standard deviation of 0.79. The aspect most appreciated by the students was the statement “It is my responsibility as a student to learn what I need to know from this simulation activity”, with 66% of the students indicating total agreement. This was followed by the statement “I know how to get help when I do not understand the concepts covered by this simulation”, with 63% of the students indicating total agreement. The aspect least valued by the students was the statement “It is the teacher’s responsibility to tell me what I need to learn from the content of the simulation activity during class time” with a mean score of 4.35 and a standard deviation of 0.99. Further details about the levels of satisfaction and self-confidence in student learning in scenario 1 are presented in Table 2.

Table 3 shows the means of the instrument scores among the variables sex, age, and previous preparation time with the levels of satisfaction and self-confidence of the students in scenario 1 (family assessment). The results revealed a statistically significant difference for previous preparation time, indicating that students who dedicated more study time prior to the simulation experience achieved higher levels of satisfaction (*p* = 0.002) and self-confidence (*p* = 0.001).

Furthermore, in the satisfaction construct, a statistically significant result was found in the comparison by sex (*p* = 0.040). Although other comparisons did not exhibit a statistically significant association with students’ satisfaction and self-confidence, a mean difference was observed between male ($\bar{x}$ = 60.92, SD = 3.89) and female ($\bar{x}$ = 56.95, SD = 10.91) students in the total instrument score.

### 3.3. Scenario 2 (Family Intervention)

In scenario 2 (family intervention), the mean total score of the instrument was 59.78 (range: 13–65), with a standard deviation of 7.49. For the satisfaction construct, the mean was 4.60, with a standard deviation of 0.58. The most notable aspect was the positive evaluation of the item stating “The teaching methods used in this simulation proved to be helpful and effective”, with 72% of the students expressing full agreement with this statement. This was followed by the statement “The simulation provided me with a variety of teaching materials and activities that enhanced my learning in the clinical curriculum”, with 67% of students strongly agreeing with this statement. The aspects least valued by the students was the statement “I enjoyed how the simulation was taught”, “The teaching materials used in this simulation were very motivating and helped me learn”, and “The way my teacher(s) taught in the simulation suited my way of learning”, with a mean score of 4.57 with a standard deviation of 0.67, 0.66, and 0.69, respectively.

Regarding the self-confidence construct, the mean was 4.59, with a standard deviation of 0.58. The item with the best evaluation from the students was “I am confident that I will have mastery of the content of the simulation activity presented to me by my instructors”, with 71% of the students expressing total agreement with this statement. This was followed by the items “I am confident that with this simulation I will develop the skills and obtain the necessary knowledge to carry out the tasks in the clinical setting” and “It is my responsibility as a student to learn what I need to know from this simulation activity”, both with 69% of the students expressing total agreement with these statements. The aspect least valued by the students was the statement “It is the teacher’s responsibility to tell me what I need to learn from the content of the simulation activity during class time”, with a mean score of 4.44 and a standard deviation of 0.85. Further details about the levels of satisfaction and self-confidence in student learning in scenario 2 are presented in Table 4.

Table 5 shows the means of the instrument scores according to the variables sex, age, and previous preparation time with the levels of satisfaction and self-confidence of the students in scenario 2 (family intervention). Notably, investing more study time prior to the simulation experience (mean $\bar{X}$ = 56.86, SD = 7.491) compared to spending less time (mean $\bar{X}$ = 52) had a positive impact on the mean scores of the instrument. Despite these observed differences, a statistically significant difference was not found.

It is important to highlight that the comparison of the means for the variable of preparation time prior to practice could not be conducted in this scenario, as only one participant reported not having done any prior preparation. Additional results are detailed in Table 5.

## 4. Discussion

Our study aimed to describe the levels of satisfaction and self-confidence among nursing students at the University of Jaén, Spain, following their participation in CS experiences as part of the Family and Community Nursing III course. Across both of the scenarios analyzed, the nursing students expressed a high degree of satisfaction and self-confidence regarding the CS activity.

In scenario 1 (family assessment), the students exhibited higher levels of self-confidence compared to satisfaction with the CS. Conversely, in scenario 2 (family intervention), the mean scores were nearly identical for both constructs.

Preparation time prior to simulation practice demonstrated a statistically significant association with the total score in scenario 1 (family assessment), with scores increasing with longer preparation times. Additionally, a statistically significant association was found between gender and the satisfaction construct. These findings underscore the effectiveness of CS in enhancing students’ satisfaction and self-confidence in nursing education, while also highlighting the importance of adequate preparation time and considering gender differences in educational experiences.

The results obtained indicate a notably high level of satisfaction among participants for both CS scenarios. This elevated satisfaction can be attributed to factors such as the quality and realism of the simulation environment. A well-designed simulation environment that accurately mirrors real clinical situations has been shown to foster higher participant commitment and satisfaction [25].

When comparing our results with previous research, it is evident that our satisfaction scores surpass those reported in other studies. For instance, a study conducted in Brazil involving 52 nursing students in a semiology and semiotechnology course revealed a mean satisfaction of 4.18 among the participants [17]. Similarly, two studies involving Saudi nursing students who engaged in CS reported mean satisfaction scores of 3.76 and 4.60 among groups of 76 and 80 participants, respectively [28,29]. Another study by Lubbers and Rossman [30], which used a quasi-experimental design with 61 pediatric community simulation nursing students, reported a mean satisfaction score of 4.10.

These comparisons suggest that our teaching strategies and simulation design are highly effective in creating a satisfying experience for nursing students. Furthermore, our results closely align with those obtained in a controlled clinical trial involving 34 nursing students across three scenarios, which reported a mean satisfaction score of 4.65. This further underscores the quality and effectiveness of our teaching practices in the context of CS [18].

In both scenarios, our findings regarding the construct of self-confidence during CS stand out compared to previous research. Our mean scores exceeded those reported in previous studies, where scores ranging from 3.70 to 4.46 were observed in various simulation contexts [17,18,28,29,30].

These remarkable results in self-confidence could be attributed to several factors. Firstly, the design of realistic scenarios plays a crucial role, as it enables students to authentically engage with the simulation experience. Additionally, the provision of feedback (debriefing) during the simulation sessions and the continuous support offered by the teachers contribute significantly to enhancing students’ self-confidence.

A comparison with previous research [17,18,28,29,30] suggests that our teaching strategy may be highly effective in fostering students’ self-confidence, which in turn could have a positive impact on their professional performance. This is probably due to the fact that our didactic strategy also included a pre-recorded video (Supplementary Material) that provides a significant enhancement to our results. This resource clearly illustrates the challenges students will encounter in their simulation experience, likely contributing to a deeper understanding and greater clarification of the hurdles they will need to overcome.

Our findings revealed statistically significant correlations between gender and satisfaction, as well as between previous preparation time and both constructs (satisfaction and self-confidence) in scenario 1 (family assessment). However, no significant correlations were found between the other variables in either scenario 1 or scenario 2. These results align with previous research that also found no associations between students’ sociodemographic data and clinical simulations [28,31].

Although significant associations were not identified in some variables, our results reinforce the need to consider diverse teaching strategies that adapt to the individual characteristics of students. For example, there was a tendency for higher scores among male students, those under 24 years of age, and those who spent more hours on pre-preparation. This suggests the importance of addressing possible differences in the way students approach and feel in simulated clinical scenarios. For future research, it would be interesting to consider developing preparation strategies that also address the emotional and psychological factors that influence students’ confidence and satisfaction during clinical simulations.

In our research, we observed a gap in previous studies regarding the evaluation of the constructs of satisfaction and self-confidence in nursing students within the context of family and community nursing. However, the existing research consistently demonstrated the benefits of CS for the development of students’ clinical reasoning and practical competencies [32,33].

Our findings are in line with the comprehensive review conducted by Cant and Cooper [6] in 2017, which focused on identifying, evaluating, and synthesizing the evidence on the impact of simulation-based education in nursing. Analyzing 25 reviews covering more than 700 primary studies, the authors concluded that simulation experiences significantly contribute to improving nursing students’ knowledge, acquisition of clinical skills, self-efficacy, confidence, and competence.

Furthermore, we did not find research conducted in Spanish that used the NLN scale [25], despite its widespread use in international studies [17,18,30,34]. One possible explanation for this discrepancy could be the recent validation of the instrument in Spanish, which may account for its limited use in previous studies [26].

In terms of limitations, it is crucial to acknowledge that our cross-sectional descriptive design lacks the ability to establish causal relationships due to the absence of temporal follow-up. This limitation means we cannot infer causality between variables. While significant associations were not found for certain variables, the lack of correlation may be attributable to the specific nature of the sample. It is possible that factors not accounted for in this study could influence the relationships between the analyzed variables. Additionally, it is important to note that this study relied on a convenience sample, due to the accessibility to the students who took the subject involved in the use of CS. The non-random selection of participants may have introduced biases into the sample, potentially limiting the generalizability of the results to a broader population. By acknowledging these limitations, we can provide a clearer understanding of the scope and implications of our study’s findings.

For future research, it would be advisable to replicate this study using a larger sample size obtained through probability sampling methods. By doing so, we can enhance the generalizability of the results to a broader population of nursing students. Additionally, it would be pertinent to explore other aspects of learning, such as academic grades, critical thinking abilities, motivation levels, and prior knowledge, among nursing students at different academic levels. Investigating these factors can provide a more comprehensive perspective on the effects of CS on the development of skills and knowledge throughout the academic training of nursing students. By addressing these aspects in future research endeavors, we can gain deeper insights into the impact of CS on nursing education and further optimize educational practices in this field.

## 5. Conclusions

Our research demonstrated that nursing students exhibit significantly higher levels of satisfaction and self-confidence following their participation in clinical simulation experiences involving family assessment and intervention scenarios. Importantly, adequate preparation time prior to the simulation practice was found to be significantly associated with heightened levels of satisfaction and self-confidence, underscoring the importance of thorough preparation in enhancing the student experience.

Furthermore, correlations were identified between gender and satisfaction in the family assessment scenario, suggesting potential areas for further exploration. The quality and realism of the simulation, along with the design of well-structured scenarios, likely played pivotal roles in fostering a positive experience for students, promoting their engagement and satisfaction. The use of CS has emerged as a highly effective educational strategy for nursing students, especially in subjects such as family and community nursing, where there is the promotion of self-care with interventions at the family level. This study fills a significant gap in the research by examining the perception of satisfaction and self-confidence among nursing students in the context of family and community nursing, offering promising results for the design of future educational programs and the continuous improvement of the quality of nursing education.

These findings contribute to our understanding of the impact of clinical simulation on nursing education and provide valuable insights for educators seeking to enhance the learning experiences of nursing students.

## Figures and Tables

**Table 1:** Sociodemographic characteristics of participants.

	N (%)	M (±SD)	Range
**Scenario 1 (N = 96)**			
Age		24.05 (7.49)	19–51
Sex			
Male	12 (13)		
Female	84 (87)		
Preparation time prior to practice			
I spent less than 2 h of study time	25 (26)		
I spent between 2 and 4 h of study	47 (49)		
I spent between 4 and 6 h of study	16 (16)		
I spent more than 6 h of study time	5 (5)		
I did not have time to do pre-practice preparation	3 (3)		
**Scenario 2 (N = 97)**			
Age		23.63 (7.57)	19–55
Sex			
Male	12 (13)		
Female	85 (87)		
Preparation time prior to practice			
I spent less than 2 h of study time	11 (11)		
I spent between 2 and 4 h of study	46 (48)		
I spent between 4 and 6 h of study	29 (30)		
I spent more than 6 h of study time	10 (10)		
I did not have time to do pre-practice preparation	1 (1)		

M: mean; SD: standard deviation.

**Table 2:** Descriptive statistics for scenario 1 of the assessment items of the Student Satisfaction and Self-Confidence with Learning Scale (N = 96).

	M (SD)	1 n (%)	2 n (%)	3 n (%)	4 n (%)	5 n (%)
**Total for instrument**	57.45 (10.37)					
**Satisfaction construct**	4.38 (0.85)					
The teaching methods used in this simulation were helpful and effective.	4.33 (1.01)	4 (4)	2 (2)	8 (8)	26 (27)	56 (58)
The simulation provided me with a variety of teaching materials and activities that furthered my learning in the clinical curriculum.	4.48 (0.83)	2 (2)	1 (1)	6 (6.2)	27 (28)	60 (62)
I enjoyed how the simulation was taught.	4.39 (0.89)	3 (3)	1 (1)	6 (6)	32 (33)	54 (56)
The teaching materials used in this simulation were very motivating and helped me learn.	4.29 (0.99)	3 (3)	3 (3)	10 (10)	27 (28)	53 (55)
The way the simulation was taught in suited my way of learning.	4.41 (0.91)	4 (4)		4 (4)	33 (34)	55 (57)
**Self-confidence construct**	4.44 (0.79)					
I am confident that I will master the content of the simulation activity presented to me by my instructors.	4.44 (0.90)	4 (4)	1 (1)		35 (36)	56 (58)
I am confident this simulation covered crucial content necessary to master the clinical curriculum.	4.38 (0.99)	5 (5)		6 (6)	28 (29)	57 (59)
I am confident that with this simulation I will develop the skills and gain the knowledge necessary to perform the tasks in a clinical setting.	4.39 (0.93)	4 (4)		6 (6)	31 (32)	55 (57)
My professors used very helpful resources to teach the simulation.	4.46 (0.90)	3 (3)	2 (2)	3 (3)	28 (29)	60 (62)
It is my responsibility as a student to learn what I need to know from this simulation activity.	4.53 (0.84)	3 (3)		4 (4)	25 (26)	64 (66)
I know how to ask for help when I do not understand the concepts covered by this simulation.	4.53 (0.80)	3 (3)		1 (1)	31 (32)	61 (63)
I know how to use the simulation activities to learn crucial aspects of these skills.	4.48 (0.83)	3 (3)		3 (3)	32 (33)	58 (60)
It is the teacher’s responsibility to tell me what I need to learn from the content of the simulation activity during class time.	4.35 (0.99)	5 (5)	1 (1)	3 (3)	33 (34)	54 (56)

M: mean; SD: standard deviation. 1: I strongly disagree with the statement; 2: I disagree with the statement; 3: undecided; neither disagree nor agree with the statement; 4: I agree with the statement; 5: I strongly agree with the statement.

**Table 3:** Differences in student satisfaction and self-confidence according to the variables sex, age, and previous preparation time in scenario 1 (N = 96).

**Male** **n = 12**			**Female** **n = 84**				
**Variable**	**M**	**SD**	**M**	**SD**	***p*-Value**	**Cohen’s D**	**95% CI**
Total instrument	60.92	3.89	56.95	10.91	0.217	0.38	(−)0.02; 0.79
Satisfaction construct	4.76	0.42	4.32	0.88	0.040	0.52	0.11; 0.93
Self-confidence construct	4.63	0.36	4.41	0.83	0.168	0.28	(−)0.13; 0.68
**Age ≤ 24 Years** **n = 73**			**Age > 24 Years** **n = 23**				
**Variable**	**M**	**SD**	**M**	**SD**	***p*-Value**	**Cohen’s D**	**95% CI**
Total instrument	58.08	8.9	55.43	14.12	0.485	0.26	(−)0.15; 0.66
Satisfaction construct	4.41	0.77	4.26	1.08	0.486	0.18	(−)0.23; 0.58
Self-confidence construct	4.50	0.66	4.26	1.10	0.205	0.31	(−)0.10; 0.71
**No Previous Preparation** **n = 3**			**One or More Hours of Previous Preparation** **n = 93**				
**Variable**	**M**	**SD**	**M**	**SD**	***p*-Value**	**Cohen’s D**	**95% CI**
Total instrument	37.67	19.65	58.09	9.46	0.001	2.09	1.58; 2.59
Satisfaction construct	2.86	1.28	4.42	0.80	0.002	1.92	1.43; 2.40
Self-confidence construct	2.91	1.65	4.49	0.71	0.001	2.13	1.62; 2.63

M: mean, SD: standard deviation, CI: confidence interval. *p* value from Student’s *t*-test.

**Table 4:** Descriptive statistics for scenario 2 of the assessment items of the Student Satisfaction and Self-Confidence with Learning Scale (N = 97).

	M (SD)	1 n (%)	2 n (%)	3 n (%)	4 n (%)	5 n (%)
**Total for instrument**	59.78 (7.49)					
**Satisfaction construct**	4.60 (0.58)					
The teaching methods used in this simulation were helpful and effective.	4.68 (0.60)	1 (1)		1 (1)	25 (26)	70 (72)
The simulation provided me with a variety of teaching materials and activities that furthered my learning in the clinical curriculum.	4.62 (0.63)	1 (1)		2 (2)	29 (30)	65 (67)
I enjoyed how the simulation was taught.	4.57 (0.67)	1 (1)		4 (4)	30 (31)	62 (64)
The teaching materials used in this simulation were very motivating and helped me learn.	4.57 (0.66)	1 (1)		3 (3)	32 (33)	61 (63)
The way my teacher(s) taught in the simulation suited my way of learning.	4.57 (0.69)	1 (1)	1 (1)	2 (2)	31 (32)	62 (64)
**Self-confidence construct**	4.59 (0.58)					
I am confident that I will have mastery of the content of the simulation activity presented to me by my instructors.	4.66 (0.62)	1 (1)		2 (2)	25 (26)	69 (71)
I am confident that this simulation has covered critical content necessary for mastery of the clinical curriculum.	4.61 (0.65)	1 (1)		3 (3)	28 (29)	65 (67)
I am confident that with this simulation I will develop the skills and gain the knowledge necessary to perform the tasks in the clinical setting.	4.62 (0.66)	1 (1)		4 (4)	25 (26)	67 (69)
My professors have used very helpful resources to teach the simulation.	4.63 (0.63)	1 (1)		2 (2)	28 (29)	66 (68)
It is my responsibility as a student to learn what I need to know from this simulation activity.	4.63 (0.65)	1 (1)		3 (3)	26 (27)	67 (69)
I know how to get help when I do not understand the concepts covered by this simulation.	4.60 (0.64)	1 (1)		2 (2)	31 (32)	63 (65)
I know how to use the simulation activities to learn critical aspects of these skills.	4.60 (0.64)	1 (1)		2 (2)	31 (32)	63 (65)
It is the teacher’s responsibility to tell me what I need to learn from the content of the simulation activity during class time.	4.44 (0.85)	1 (1)	3 (3)	8 (8)	25 (26)	60 (62)

M: mean; SD: standard deviation. 1: I strongly disagree with the statement; 2: I disagree with the statement; 3: undecided; neither disagree nor agree with the statement; 4: I agree with the statement; 5: I strongly agree with the statement.

**Table 5:** Differences in the student satisfaction and self-confidence according to the variables sex, age, and previous preparation time in scenario 2 (N = 97).

**Male** **n = 12**			**Female** **n = 85**				
**Variable**	**M**	**SD**	**M**	**SD**	***p*-Value**	**Cohen’s D**	**95% CI**
Total instrument	61.08	4.92	59.6	7.79	0.524	0.20	(−)0.21; 0.60
Satisfaction construct	4.76	0.37	4.57	0.61	0.298	0.32	(−)0.08; 0.73
Self-confidence construct	4.65	0.42	4.58	0.60	0.715	0.12	(−)0.28; 0.52
**Age ≤ 24 Years** **n = 79**			**Age > 24 Years** **n = 18**				
**Variable**	**M**	**SD**	**M**	**SD**	***p*-Value**	**Cohen’s D**	**95% CI**
Total instrument	60.13	7.53	58.28	7.34	0.348	0.25	(−)0.16; 0.65
Satisfaction construct	4.62	0.60	4.47	0.53	0.332	0.26	(−)0.15; 0.66
Self-confidence construct	4.62	0.58	4.48	0.60	0.372	0.24	(−)0.16; 0.64

M: mean; SD: standard deviation; CI: confidence interval. *p* value from Student’s *t*-test.

## Data Availability

All data are available from the authors upon reasonable request.

## Supplementary Materials

The following supporting information can be downloaded at https://www.mdpi.com/article/10.3390/nursrep14020078/s1.
