# Supplementary material for: Evaluating Satisfaction and Self-Confidence among Nursing Students in Clinical Simulation Learning
Source: Nurs Rep. 2024 Apr 25;14(2):1037–48. doi: 10.3390/nursrep14020078 (PMC11130807; doi:10.3390/nursrep14020078)
Supplement: Supplementary file 1 [file nursrep-14-00078-s001.zip › nursrep-2936419-supplementary.pdf]

nursing reports

Supplementary information

Evaluating satisfaction and self-confidence among nursing students in clinical simulation learning

Sara Moreno-Cámara [1], Henrique da-Silva-Domingues [2*], Laura Parra-Anguita [3] and Belén Gutiérrez-Sánchez [4]

[1] University of Jaen; smcamara@ujaen.es
[2] University of Jaen; hda@ujaen.es
[3] University of Jaen; lparra@ujaen.es
[4] University of Jaen; bgutierr@ujaen.es
* Correspondence: hda@ujaen.es

In this document, the authors explain in more detail the use of clinical simulation (CS) used in this research.

Family and Community Nursing III (EFyC III) course aims to contribute to the training of future graduates in Nursing in the field of community health and in the primary health care. Among the expected outcomes for EFyC III we highlight that students will be able to:

- Promote the participation of individuals, families and groups in their health and illness process. Identify factors related to health and environmental problems, in order to attend to people in situations of health and illness as members of a community. Identify and analyse the influence of internal and external factors on the level of health of individuals and groups. Educate, facilitate and support the health and well-being of community members whose lives are affected by health problems, risk, suffering, illness, disability or death.
- Have the ability to assess family structure and dynamics and to plan, deliver and evaluate care for family and caregivers considering family and community resources.

Clinical Simulation Scenarios

At the curricular level, nursing students engage in subjects related to family and community nursing throughout their training. These subjects aim to equip future professionals with a broad spectrum of knowledge, skills, and abilities to provide care for individuals, families, and communities. Specifically, in the Degree in Nursing program at the University of Jaén, Spain, the EFyC III course focuses on the promotion of self-care through interventions at the family level.

The high-fidelity simulation strategy employed to train nursing students in family intervention was based on the Nursing Process. This approach systematically organizes nursing care for patients and encompasses five phases: assessment, diagnosis, planning, implementation (intervention), and evaluation. In the context of CS, the specific phases addressed were the family assessment phase (scenario 1) and the implementation phase (family intervention) (scenario 2).

The simulation experience was conducted in a seminar setting designed to resemble a home environment. The scenario involved the participation of the senior theater group of the University of Jaén, portraying a family caring for an elderly dependent relative. The script followed by the actors was meticulously directed by the professors of the course.

For the CS, four face-to-face practical sessions were dedicated, with two sessions allocated to scenario 1 and two sessions to scenario 2. Each practical session lasted three hours and accommodated 14 students. During these sessions, the entire group participated in pre-briefing and debriefing sessions for both scenarios

Simultaneously, at a theoretical level, the nursing intervention in the family setting was taught. During this period, students were required to conduct a family assessment (scenario 1) and a subsequent family intervention (scenario 2) based on the assessment findings. Students were provided with guidelines outlining the objectives and tasks for the practical sessions related to nursing intervention in the family setting.

The Degree in Nursing at the University of Jaen has two groups of students in the third year (one in the morning and the other in the afternoon) in which the same contents are taught. In total, 130 students attend the Family and Community Nursing III course. The practical programme is carried out in mini-groups. Each group (morning and afternoon) is attended by about 65 students who are divided into 5 groups, i.e. each practical group consists of approximately 14 students. The teacher repeats the exercises 10 times.

In order to carry out the simulations, students must have previously attended the theoretical classes related to the scenarios addressed, with the purpose of preparing them and developing the cases. The theoretical foundations taught are linked to nursing intervention in the family setting, including topics such as family and health, family structure and dynamics, problems in the different stages of the family cycle, home care methodology, assessment of family health, dysfunctional patterns, nursing diagnoses in the family setting, safety and prevention of accidents in the home, basic aspects of family intervention, social resources to support families, technical aids for home care, characteristics and consequences of family care, assessment of the family caregiver, and support and care of the family caregiver. In addition, before the CS, students have access to a series of pre-recorded videos by the teachers of the subject, where they can observe the two scenarios of the CS (family assessment and family intervention) that students will have to face. The pre-recorded videos, together with all the course materials, are available on the platform where the course is hosted.

Scenario 1 (family assessment)

For Scenario 1, students carried out the CS between 23 and 25 October 2023.

Prior to the CS, students were advised to review their previous courses on clinical interviewing and nursing assessment, with special attention to assessment using Marjory Gordon's Functional Patterns, as well as the main instruments, tests, and scales available for assessing different functional.

On the day of the simulation, each student performed a role-play on family assessment (with the participation of the theatre group of the University of Jaen), using as a guide the "Guide for family health assessment and recording" provided by the teachers. Each student assumed the role of a nurse conducting the family assessment on a family that was present in the classroom (set up as a home). The teacher indicated which part of the interview-assessment each student would carry out and assessed the student's skills in terms of: interview technique: verbal and non-verbal communication, empathy, active listening, bidirectionality; knowledge about the structure of the "Guide for family health assessment and recording" and appropriate use of the different assessment instruments.

Scenario 2 (family intervention)

The students carried out the CS between 30 October and 08 November 2023.

Scenario 2 (family intervention) took place once the Family Health Assessment (scenario 1) had been carried out, the nursing problems/diagnoses present in that family had been identified and the corresponding care plans had been established (Scenario 1 family assessment).

This scenario consisted of each student dramatizing the development of an intervention aimed at improving or resolving one of the priority problems identified in the family assessment (scenario 1). In the CS, each student had a maximum of 15 minutes to develop their role-play of the proposed family intervention. The students had to make a brief presentation of their case to the rest of the group, justifying the reasons for selecting the diagnosis/problem on which they were going to intervene. From this moment on, the

dramatization began and they assumed the role of family nurse, having to develop the planned intervention taking into account that they were in the family home (simulation classroom set up as a home and with the participation of the drama group as the family to be intervened) and adjusting to the specific characteristics of their case. Before the practical sessions, students were tasked with planning both the assessment and the intervention for the family. They were required to submit a document to the teachers containing essential details such as a brief description of the family, the selected nursing diagnosis, the developed care plan, a description of the planned intervention along with its objectives and proposed interventions, the materials and resources to be used, and the anticipated evaluation timeline for the intervention.

With the didactic strategy employed through CS, the evaluation instruments and the levels of satisfaction and self-confidence reported in this research, we can affirm that the objectives set at the beginning of this document for the EFyC III course were fully met.
